# Cirrhosis presenting as Parkinsonism

**DOI:** 10.4103/0972-2327.42938

**Published:** 2008

**Authors:** Mohan L. Noone, V. G. Pradeep Kumar, K. Ummer, Laila Achambat, K. A. Salam

**Affiliations:** Department of Neurological Sciences, Baby Memorial Hospital, Calicut, India

**Keywords:** Acquired hepatocerebral degeneration, cirrhosis, parkinsonism

## Abstract

Cirrhosis presenting as Parkinsonism is a distinct subset of acquired chronic hepatocerebral degeneration. The entity is not rare, and unless suspected, cirrhosis can easily be overlooked. We report our experience with three such patients. They presented to us, over a period of two years, with symmetrical Parkinsonism and were later diagnosed to have cirrhosis with portal hypertension. All patients had minimal or absent tremors. Reversal of serum albumin to globulin ratio and evidence of cirrhosis on abdominal ultrasound were consistent. All three patients had the characteristic MRI abnormality of symmetrical T1 hyperintensity in basal ganglia and anterior midbrain. They improved to variable extents after treatment for cirrhosis, along with dopa agonists. We stress the importance of recognizing this syndrome and briefly review the relevant literature.

## Introduction

Association between the basal ganglia and liver disease has an interesting history. Wilson first described familial hepatolenticular degeneration, which bears his name.[1] Soon after this, von Woerkom described chronic neurological involvement in cases of advanced cirrhosis, unrelated to Wilson's disease. Many years later, this involvement was named as acquired chronic hepatocerebral degeneration (AHCD) by Victor *et al.*[2] AHCD is clinically distinct from hepatic encephalopathy, being a chronic, persistent syndrome, predominated by symptoms related to basal ganglia dysfunction.[3] Altered MRI signals from the basal ganglia have been consistently reported in AHCD.[4]

Cirrhosis presenting as Parkinsonism is a distinct subset of AHCD. The entity is not rare and, unless suspected, cirrhosis can easily be overlooked. We describe our experience with three such cases seen over a period of two years.

## Case Reports

### Case 1

**Figure 1:**
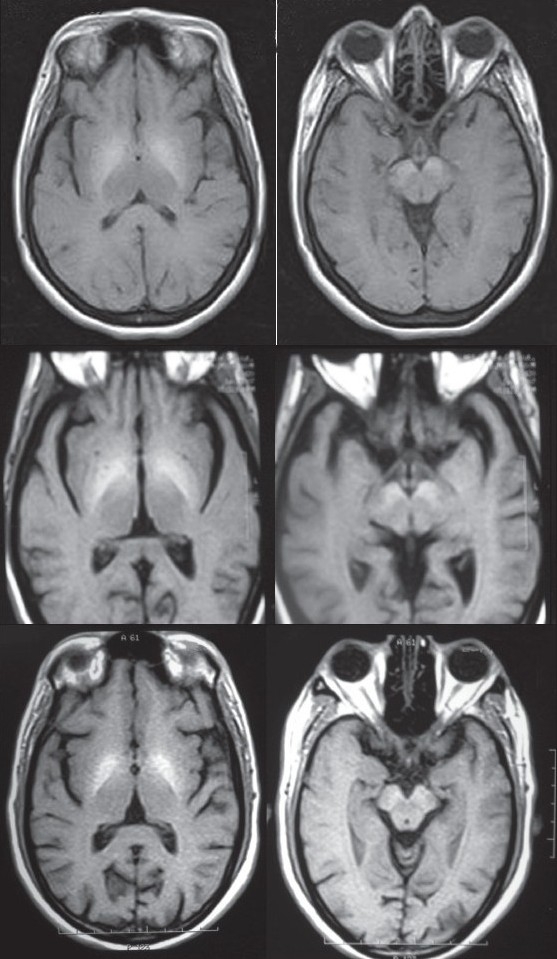
MRI Brain showing symmetrical hyperintensities in pallida and anterior midbrain in case 1 (A), case 2 (B) and case 3 (C).

A 54-year-old lady presented in September 2005 with progressive slowness of gait and tremulousness of limbs since eight months, postural instability and hypophonia since three months. There was no history of memory lapses, delirium or other neuropsychiatric disturbances. On examination, she was alert and well-oriented with a masked facial expression, bilateral cogwheel rigidity, symmetrical resting tremor in the upper limbs and moderate postural instability. No motor weakness, sensory loss, ataxia or pyramidal signs were noted. There were no corneal deposits or hepatosplenomegaly. She was diagnosed to have Parkinsonism and evaluated. MRI Brain showed symmetrical hyperintensity on T1 weighted images in the pallida and anterior midbrain [Figure 1A], which were isointense on T2 weighted images. Liver function tests showed subtle derangements and A/G reversal: Total bilirubin 1.8mg/dl, ALT 22U/L, AST 41U/L, Alkaline Phosphatase 73 U/L (all enzymes within normal limits), Serum Albumin 3.0 g/dl, Globulin 3.8g/dl A/G Ratio: 0.8. Serum Copper and Ceruloplasmin were normal. Serum Iron, Total Iron Binding Capacity (TIBC) and Serum Ferritin were normal. HBsAg and Anti HCVAb were negative. Abdominal ultrasound showed features consistent with chronic liver disease with portal hypertension. Oesophago-gastro-duodenoscopy (OGD scopy) showed congestive gastropathy with small fundal varices. Specific etiology for cirrhosis could not be found in this case (Cryptogenic).

The patient was started on treatment for cirrhosis and portal hypertension with lactulose, ornithine aspartate and propranolol along with ropinirole. She showed gradual improvement and is currently able to independently perform activities of daily living (ADL).

### Case 2

A 60-year-old man presented in June 2006 with slowness of movements, difficulty in walking and rigidity of limbs of gradual onset, since two years, with occasional tremors of the upper limbs. There was no history of cognitive decline, falls or incontinence. Examination revealed symmetrical Parkinsonism with minimal tremors in the upper limbs. A clinical diagnosis of Parkinsonism was made. Carbidopa-levodopa was started (up to 75+300 mg/day), along with ropinirole (1.5 mg/day). Symproms of Parkinsonism improved initially, but later worsened. Three months later, entocapone (600 mg/day) was also added, with little clinical improvement.

One year after presentation, an MR imaging of his brain was done, considering his lack of response to standard medication. Abnormal T1 hyperintense signals were noted in the pallida and anterior midbrain bilaterally [Figure 1B]. At this point, hepatocerebral syndrome was suspected and admission was advised. He was, however, lost for follow-up. Nine months later, he was bought to the emergency department, following a brief febrile illness. He was drowsy and mildly confused, with bilateral pitting pedal edema. There was no icterus, hepatosplenomegaly or ascites. Bilateral rigidity and asterixis were present. Liver functions were abnormal (Total Bilirubin 1.1 mg/dl, ALT 60U/L, AST 66U/L, Alkaline Phosphatase 179 U/L, S Albumin 3.0 g/dl, Globulin 3.3 g/dl A/G Ratio 0.9). Abdominal sonogram revealed abnormal hepatic echotexture, suggestive of cirrhosis with features of portal hypertension. OGD scopy revealed grade III oesophageal varices, with congestive gastropathy. Work up for Wilson's disease was negative, S. ferritin levels were normal and HBs Antigen was negative. HCV antibodies were positive in high titers, and HCV RNA quantitative assay (Roche-Ampiclore) showed titer of 152000 IU/ml (high). Diagnosis of HCV related cirrhosis with acquired hepatocerebral syndrome was made.

### Case 3

A 69-year-old man presented in November 2007 with difficulty in walking and imbalance since six months. He had a history of coronary artery disease and hypertension, for which he was on treatment. Physical examination findings included masked facies, normal eye movements, no Keyser-Fleischer rings, symmetrical bradykinesia and mild rigidity with no tremors. Tendon reflexes were brisk. His MRI showed symmetrical pallidal and anterior midbrain T1 hyperintensity [Figure 1C], raising suspicion of hepatocerebral syndrome. Liver functions were mildly abnormal (Total bilirubin 1.6 mg/dl, ALT 32U/L, AST 50 U/L, Alk Phosphatase 99 U/L, S Albumin 3.2 g/dl, globulin 3.6 g/dl (A/G Ratio 0.9). S Copper and Ceruloplasmin were normal. OGD Scopy showed grade II varices and USG abdomen showed hepatomegaly, with no features of portal hypertension. He is under evaluation for etiology for chronic liver disease. After treatment for hepatic decompensation and low dose of carbidopa-levodopa (75+300 mg / day), he has improved. He has never reported any episodes of encephalopathy.

## Discussion

We report three cases of cirrhosis presenting to the neurologist with Parkinsonism. All the reports we found in literature were of cirrhosis related Parkinsonism in patients already known to have liver disease. In contrast, our cases directly presented to the clinical neurologist with clinical features of Parkinsonism and no prior history of liver disease.

All the patients had a fairly rapid progression of symmetrical bradykinesia and gait impairment, with minimal or absent tremors. They had no signs of encephalopathy at presentation. (Though the second patient ultimately presented with encephalopathy, he had no such symptoms when he first presented with Parkinsonism.) Symmetric T1 hyperintensity in the pallida and anterior midbrain on MR imaging was consistently seen in all the three patients. The only laboratory abnormality common to all the patients was reversal of the A/G ratio – an important clue, though easily overlooked in a patient of Parkinsonism, unless a diagnosis of hepatocerebral syndrome is suspected.

In the largest study available in literature on chronic Parkinsonism associated with cirrhosis, Burkhard *et al.* report their findings in 11 patients, seen over a period one year.[3] They were recruited from a hepatic transplantation unit. Progression from onset to peak of Parkinsonism in these patients was rapid (mean of 7.2 months). Global slowness of movements and gait impairment were the main initial symptoms. Notably, none of the patients had resting tremor; postural tremor was seen in all patients. None had evidence of dementia. MRI in all patients showed, on T1-weighted images, bilateral and symmetrical hyperintensities that were restricted to the substantia nigra and the globus pallidus.

All our patients had identical MRI abnormalities and comparable clinical presentations. Thus they conform to the description of Burkhard *et al.* The cause for the T1 hyperintensity on MRI is recognized to be due to manganese deposition.[5] Deposition in the anterior midbrain in the region of the pars compacta of substantia nigra (SNPC) probably correlates with the development of clinical Parkinsonism.[3] Thus, MR evidence of SNPC injury is seen in most cases of cirrhosis related Parkinsonism. Interestingly, it has been suggested that porto-systemic shunting, rather than hepatocellular dysfunction, is responsible for manganese accumulation and damage to basal ganglia.[6]

Thus, there is a fair consensus in literature that the pathophysiology of AHCD involves manganese accumulation in the basal ganglia as well as SNPC, which in turn is primarily due to increased porto-systemic shunting. There is also some evidence that deposition in the SNPC may specifically correlate with clinical development of Parkinsonism. One may thus hypothesize that cirrhosis patients who present with Parkinsonism may have significant early SNPC involvement. However, why these features occur only in a subset of patients with cirrhosis is currently not clear. Bradykinesia in cirrhotics has also been linked to abnormal movement initiation, and altered metabolism in frontomesial cortical areas has been demonstrated.[7] The relative importance of these different mechanisms warrants further exploration.

Burkhard *et al.* mention in the article that the frequency of Parkinsonism related to cirrhosis is probably underestimated. Our experience of seeing three patients over a period of two years in a community based neurology center also suggests that this entity is not rare. Thus, it is important to suspect hepatocerebral Parkinsonism when a middle-aged patient presents with the rapid onset of symmetric Parkinsonism, with mild or absent tremor. The diagnosis can be made fairly certain by demonstrating A/G reversal, the characteristic MRI abnormalities and abdominal sonological features consistent with cirrhosis. Patients improve to variable extents after treatment for cirrhosis, along with dopa agonists. It is reported in literature that hepatocerebral Parkinsonism can resolve after successful treatment for porto-systemic shunting[8] and liver transplantation.[9]

These patients require long term follow-up to assess their clinical outcome and survival.
